# Mapping the decision pathways of acute infection management in secondary care among UK medical physicians: a qualitative study

**DOI:** 10.1186/s12916-016-0751-y

**Published:** 2016-12-12

**Authors:** Timothy Miles Rawson, Esmita Charani, Luke Stephen Prockter Moore, Bernard Hernandez, Enrique Castro-Sánchez, Pau Herrero, Pantelis Georgiou, Alison Helen Holmes

**Affiliations:** 1 National Institute for Health Research, Health Protection Research Unit in Healthcare Associated Infections and Antimicrobial Resistance, Imperial College London, Hammersmith Campus, Du Cane Road, London, W12 0NN UK; 2 Imperial College Healthcare NHS Trust, Hammersmith Hospital, Du Cane Road, London, W12 0HS UK; 3 Department of Electrical and Electronic Engineering, Imperial College London, South Kensington Campus, London, SW7 2AZ UK; 4 Health Protection Research Unit in Healthcare Associated Infections & Antimicrobial Resistance, Hammersmith Hospital, Du Cane Road, London, W12 0NN UK

**Keywords:** Antimicrobial stewardship, Sepsis, Antibiotics, Prescriber, Grounded-theory

## Abstract

**Background:**

The inappropriate use of antimicrobials drives antimicrobial resistance. We conducted a study to map physician decision-making processes for acute infection management in secondary care to identify potential targets for quality improvement interventions.

**Methods:**

Physicians newly qualified to consultant level participated in semi-structured interviews. Interviews were audio recorded and transcribed verbatim for analysis using NVIVO11.0 software. Grounded theory methodology was applied. Analytical categories were created using constant comparison approach to the data and participants were recruited to the study until thematic saturation was reached.

**Results:**

Twenty physicians were interviewed. The decision pathway for the management of acute infections follows a Bayesian-like step-wise approach, with information processed and systematically added to prior assumptions to guide management. The main emerging themes identified as determinants of the decision-making of individual physicians were (1) perceptions of providing ‘optimal’ care for the patient with infection by providing rapid and often intravenous therapy; (2) perceptions that stopping/de-escalating therapy was a senior doctor decision with junior trainees not expected to contribute; and (3) expectation of interactions with local guidelines and microbiology service advice. Feedback on review of junior doctor prescribing decisions was often lacking, causing frustration and confusion on appropriate practice within this cohort.

**Conclusion:**

Interventions to improve infection management must incorporate mechanisms to promote distribution of responsibility for decisions made. The disparity between expectations of prescribers to start but not review/stop therapy must be urgently addressed with mechanisms to improve communication and feedback to junior prescribers to facilitate their continued development as prudent antimicrobial prescribers.

## Background

The growing threat of antimicrobial resistance (AMR) is a leading patient health and safety issue, with estimates that AMR will be responsible for more than 10 million deaths by 2050 [1]. A major driver of AMR has been the misuse of antimicrobials in humans [2]. Whilst reasons for the misuse of antimicrobials in humans are complex and multifaceted, a number of factors have been described and investigated. At the individual level, prescribers often prioritise the management of the patient in front of them, paying little regard to the long-term consequences (on future patients and generations) of overusing antimicrobials [3]. Moreover, the majority of antimicrobial prescribing is performed by individuals who are not experts in infection management and may have limited understanding of antimicrobials and AMR [2, 4–6]. At the hospital/team level a number of barriers to the effective use of antimicrobials have been described, including the role of team hierarchies and prescribing etiquette, which can often hinder external interventions to optimise prescribing behaviours [7–9]. Finally, the role of patient involvement in the decision-making process for antimicrobial prescribing is now recognised to also shape the decisions made by physicians, with patient expectations and understanding of antimicrobials being important in shaping the appropriate use of therapy during infection management [10–12].

To address the challenges posed by AMR, the importance of behaviour change interventions in improving the long-term use of antimicrobials in infection management has been recognised [8, 9, 13]. Despite the growing body of evidence describing knowledge, attitudes and cultural determinants of antimicrobial prescribing [4, 7, 14], very little data exists mapping the clinicians decision pathway for the management of infections and antimicrobial prescribing within secondary care. A greater understanding of the decision pathways taken by prescribers may allow for the development of targeted interventions for specific aspects of this pathway.

We report a study to map the decision-making process of medical physicians in secondary care for acute infection management and investigate the factors that may hinder or facilitate the effective use of antimicrobials.

## Method

### Participant recruitment

The sampling frame for this study included all non-infection specialist medical physicians (defined as either (1) clinical specialties who practiced general internal medicine, such as cardiology, respiratory, and geriatric medicine, or (2) augmented care specialties such as haematology and nephrology) who were, at the time of the study, practicing at Imperial College NHS Healthcare Trust. The Trust comprises of three separate hospitals (1500 beds) that serve a population of 2.5 million citizens. Medical physicians from those in training (i.e. on rotation and specialist trainees) to consultant grade were included. Given that the majority of UK antimicrobial prescribing is performed by physicians, we elected to exclude other healthcare professionals involved in infection management (e.g. pharmacists and nurses). Primary care physicians, surgeons, intensive care specialists and focused specialties, such as psychiatry, were excluded from this study as the focus was the management of acute infections in the medical specialty, outside of highly specialised settings. Furthermore, many specialist areas excluded tend to also engage in a broader range of antimicrobial prescribing activities (e.g. prophylactic therapy in surgery) and also often rely on support through multi-disciplinary management of infections with medical and/or infection team input, which has been demonstrated to improve patient outcomes for infection management in these settings [15–21].

Using purposive sampling, physicians were invited to participate in this study [22, 23]. The aim of this study was to map out and compare the decision-making processes employed for acute infection management on the hospital wards by non-infection medical specialties and explore any factors that influenced this process. Participants were purposively sampled at different levels of training (on-rotation, specialist trainee and consultant) with deliberate selection that aimed to reflect the diversity of medical specialties within the hospital environment. To achieve this, physicians in the 11 major non-infection medical specialties within the hospitals, who are responsible for in-patients, were contacted via email and invited to participate in face-to-face semi-structured interviews. Two follow-up emails were sent if there was no reply from the initial invitation email at weekly intervals. Respondents who accepted to participate via email were stratified into on-rotation, specialist trainee and consultant physicians for interviews. All participants consented to participating in the study and have their interviews recorded. Interviews were conducted between August 2015 and April 2016, by one researcher (TMR; a junior doctor/clinical researcher not working within the hospitals in question). A standardised, piloted 10-question semi-structured interview guide (Appendix) was initially used to structure the interviews. Participants from each of the specialties and grades of clinician were interviewed [22–24]. Interviews were continued for each stratified grade and specialty until saturation was reached and no new themes were found to emerge [22, 23, 25, 26]. All data were anonymised with only the interviewer knowing participant identities. The interviews were audio recorded and then transcribed verbatim.

The study protocol was reviewed by the West London Regional Ethics Committee and considered to meet criteria for monitoring under service evaluation governance structures (REC 15/LO/1269/ICHNT Service Evaluation SE113).

### Data analysis

A grounded theory approach was applied to data analysis [22, 23]. NVIVO Pro 11.0 software was used to support analysis of the transcripts with the same researcher (TMR) reviewing all transcripts and performing initial line-by-line coding. During analysis, emerging themes and theories were discussed with a multi-professional team of researchers including non-medical researchers (BH, PH, PG), physicians (LSPM, AH), nurses (ECS), pharmacists (EC) and social science researchers (ECS, EC) to increase reflexivity and allow the main reviewer to be more aware of their own perceptions [27]. Deviant statements that may contradict emerging themes were also actively sought out to improve the rigor of our analysis [25, 28].

## Results

Thirty four physicians from 10 non-infection medical specialties responded to the invitation email agreeing to participate in the study. However, saturation was reached after 20 interviews. Seven participants were on-rotation physicians (from newly qualified to fourth year in training), four were specialist trainees, and nine were consultant level (Table 1). The interviews ranged in duration from 12 to 32 minutes, with a median length of 20 minutes.

**Table 1:** Characteristics of medical physician participants interviewed from Imperial College Healthcare NHS Trust about their approach to infection management within secondary care

Grade	Specialty
Consultant	Acute Medicine
Consultant	Acute Medicine/Endocrinology
Consultant	Haematology
Consultant	Haematology
Consultant	Haematology
Consultant	Care of the Elderly
Consultant	Gastroenterology/Acute Medicine
Consultant	Respiratory Medicine
Consultant	Respiratory Medicine
Specialist Registrar	Care of the Elderly
Specialist Registrar	Care of the Elderly
Specialist Registrar	Cardiology
Specialist Registrar	Clinical Pharmacology & Therapeutics/General Medicine
Core Trainee Year 2	On rotation - Acute Medicine
Core Trainee Year 1	On rotation - Acute Medicine/Stroke Medicine
Foundation Year 2	On rotation - Gastroenterology
Foundation Year 2	On rotation - Respiratory Medicine/General Medicine
Foundation Year 2	On rotation - Acute Medicine
Foundation Year 1	On rotation - Renal Medicine
Foundation Year 1	On rotation - Acute Medicine

### Mapping the decision making process

**Fig. 1:**
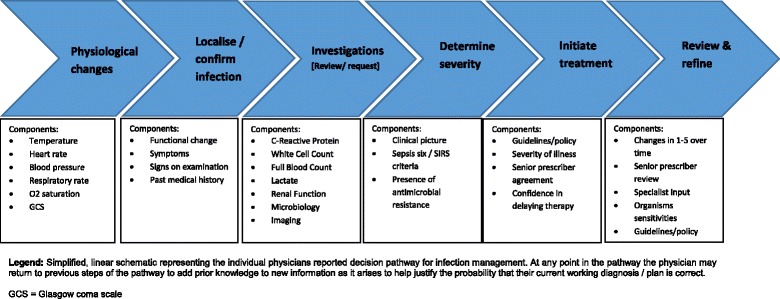
Reported individual decision pathway of infection management for medical physicians within secondary care

Analysis of the data identified six common themes describing the stages of the decision-making process for infection management. Clinicians reported that they begin with a predefined risk of an infection being present and then systematically add further information in a stepwise process, allowing optimisation of decisions on diagnosis and management in a dynamic manner. Although this process could also be viewed as a cyclical process, with physicians returning to step 1 every time they re-assess the patient, the steps and common variables considered within each step by the individual physician have been mapped out in a linear fashion for simplicity in Fig. 1.

The antibiotic decision-making process begins by looking for changes in the patient’s physiological parameters, with temperature being an important factor assessed at this point. Following this, participants report that the second stage involves attempting to localise and confirm that infection is present. This was reported to involve both searching for reported symptoms and backing this up with signs on examination. The third step reported as part of this process was the review and planning of further investigations, with C-reactive protein regarded as a key biological indicator of infection during this phase of management. Fourthly, through comparison and synthesis of findings from steps one to three, physicians reported that this allows them to construct a picture of the severity of the infection that they are managing. This was widely reported to be judged based on the overall ‘clinical picture’ that is built up during steps one to three with junior physicians also tending to report using criteria such as the ‘septic six’ or ‘Systemic Inflammatory Response Syndrome criteria’ to help determine the severity and whether or not this is classified as sepsis. The fifth reported step in the pathway is the decision of initiating antimicrobial treatment. The local microbiology guidance (written or electronic) provided within the hospitals involved was a key factor determining what therapy would be commenced, with physicians describing how steps one to four determine how this information is interpreted. Deferring therapy (or ‘watch and wait’) was an option also considered by the participants. The final step in the pathway was the review and refinement stage, which can occur through two separate or overlapping routes. The first of these is internally, with the individual physician returning to the first stage of the decision pathway and assessing for changes in each stage over time, building on the information observed during their initial review. The second route is by external review, by another physician (often reported as more senior or specialist), who uses stages one to five to review and refine the management decision made by the prescriber (Table 2).

**Table 2:** Thematic construction of medical physicians’ decision pathway for the management of acute infections in secondary care

No.	Theme	Supporting quotation
1	Bayesian process	“*It’s like Bayesian model where you refine your likelihood of diagnoses based on every new quantum of information you get, so you start off with the physiological parameters, then your differential is refined based on blood results and further refined based on the microbiology.*” [Specialist Trainee, clinical pharmacology & therapeutics]
2	Physiological parameters	“*So the first thing I do is look at the vital signs and basically make sure that the patient is haemodynamically stable*” [Consultant, acute medicine] “*Bearing in mind that most patients have a multitude of different things going on and it could be an infection or it could be many other things, the temperature would be the first thing you would look at*” [Consultant, gastroenterology/acute medicine]
3	Localise/confirm infection	“*So basically history and then examine them* [the patient]*, and then reaching a diagnosis*” [On-rotation, acute medicine/stroke] “*Access the patient clinically if I suspect infection and then I would try to determine where the infection is coming from*” [Consultant, acute medicine/endocrinology]
4	Investigations	“*Determine where the infection is coming from, where the source is and take appropriate cultures and do further investigations as indicated*” [Consultant, acute medicine/endocrinology] “*The other secondary tests, such as inflammatory markers, give us an idea of whether there really is sepsis or whether there is some other pathology*” [Consultant, respiratory 1]
5	Determine severity	“*I would still want to be assessing the severity and particularly looking for evidence of sepsis*” [Consultant, respiratory 2] “*If someone is clinically well and may have an infection I probably would rather wait because they may not need antibiotics at all. It could be a viral infection. So it very much depends on the clinical picture and medical context*” [Consultant, acute medicine/endocrinology]
6	Initiate treatment	“*We use local policy guidelines, so when I am assessing a patient I am thinking – Okay where is the focus? And also, if I know where it is [the focus], what antibiotics specifically does my hospital use?*” [On-rotation, respiratory/general medicine] “*I think if it isn’t clearly in the guideline or I am not sure, if it doesn’t easily fit into the guideline, I am going to say* [to my juniors]*, okay speak to microbiology and see what they think*” [Consultant, respiratory 1]
7	Review & refine	“*Look at the patient and make sure that they are getting better, the temperature is resolving, and their clinical symptoms and signs are improving*” [Consultant, acute medicine] “*Whoever has* [initially] *seen the patient will make the initial decision on differential diagnosis and required treatment. Thereafter, it will be the consultant review who will say yes I agree or not, and does the treatment also adhere to Trust policy? So it is very algorithmic*” [Consultant, acute medicine/endocrinology]

### Factors influencing the decision-making process

There were several key themes that emerged from the participant interviews to describe factors that influence the decision-making process outlined above. Two of these were common themes that have previously been reported in the literature; hierarchical team systems and etiquette around prescribing practices [7, 29, 30]. Several previously unreported concepts were also identified within this study surrounding stopping/de-escalating therapy, the role of guidelines and microbiology advice, and feelings of responsibility for providing optimal care. Many of these factors tended to largely influence the latter half of the decision pathway, surrounding initiation and review of antimicrobial therapy.

#### Physician skills used to assess the patient

Participants described the feeling of overall responsibility of the team and, in particular, the consultant for the patient under their care. This level of responsibility was reported to drive consultants to make autonomous decisions about the management of their own patients, using previous experiences and knowledge to make subjective assessments of the state of their patient using the pathway described in Fig. 1. Whilst junior members of the team may make initial decisions about the management of patients, the consultant reported seeing themselves as the final decision maker, with their job to review and refine the decisions of junior colleagues.

This perception projects down the medical team, with specific expectations made about junior colleagues’ actions, especially those on-rotation. On-rotation doctors report that they develop their assessment and decision-making skills through clinical practice. They are usually the first individuals to respond to an unwell patient, and tend towards reliance on objective parameters, such as heart rate and temperature in the place of subjective measures such as examination findings and general impression of the patient, which take more predominance when consultants make their assessment. Furthermore, on-rotation doctors report fears of missing the septic patient. It appears that this fear of sepsis, linked with the expectation placed on juniors to prescribe antibiotics, can often lead to inappropriate views of infection management, in particular antimicrobial prescribing. This often culminates in there being an overwhelming need to commence antimicrobials as soon as possible in anyone suspected of having an infection.

#### Antibiotic prescribing as a key component of provision of optimal care

Another reported factor that influences the provision of optimal care is the fact that the patient is in hospital, which promotes the physician to need to feel they are providing optimal care for their patient regardless of whether this is evidence based or not. A theme that emerged was that physician definitions of optimal care includes the prescription of antibiotics, with intravenous often felt to be more optimal than oral for those requiring treatment in hospital (Table 3).

**Table 3:** Selected quotes surrounding participants experiences and expectations of prescribing antibiotics

No.	Quote	
Expectations of antimicrobial use		
1	“*I think I know when would be an easy enough time as a junior doctor to go, yeah, I think this warrants Tazocin, this warrants cefuroxime IV. So for some drugs I think you have a little bit more of an ease of prescribing because you’re not too worried about the downsides*”	On-rotation, acute medicine 1
2	“*So nights, I think obviously it becomes much more of a zoo doesn’t it really, so people tend to start broad spectrum agents without really looking through previous microbes and patients have a tendency to stay on that till it’s reviewed in daytime hours*”	On-rotation, acute medicine 2
3	“*If the patient is septic or something, you have to start antibiotics within your hour, Sepsis Six, but then you’re also under pressure to get the right source*”	On-rotation, acute medicine 2
4	“*Yeah, definitely in terms of how you go but I think anyone who’s done hospital medicine now sees that Tazocin is basically the port of call for most things*”	On-rotation, cardiology
5	“*When I look back at years gone past, I think I was probably quite gung-ho with antibiotics because it was the easy option because you didn’t want to get in trouble and I’m sure plenty of patients in* [region - UK] *got BenPen* [benzylpenicillin] *and Cipro* [ciprofloxacin] *when they might have lived without it. But this is a situation in which, I think, the way I’ve changed is that I tend to look at what the risks of deferring here versus not*”	Specialist registrar, cardiology
6	“*I’ve got a bit of a nice cushion from all the senior levels about even if I prescribe the wrong antibiotic, I don’t mean of course prescribing penicillin when someone’s penicillin allergic, that’s not what I mean. I mean prescribing for example flucloxacillin when it’s an E. coli bacteria, wrong bacteria, wrong antibiotic of choice or bacteria, but an antibiotic nonetheless*”	On-rotation, acute medicine 3
7	“*I think a lot of people, myself included, would say if you are admitting the patient to hospital and they have an infection severe enough to come into hospital then you should, and I know this is not what microbiologists would say, but in my mind you like to feel like you are doing something that they couldn’t have at home and that’s why you give them some intravenous antibiotics when they come into hospital with a view to stepping them down very quickly afterwards, and I think it makes everyone feel better whether it’s the patient and more significantly the doctor*”	Consultant, general internal medicine
8	“*I would not expect an SHO* [senior house officer] *to decline to give antibiotics*”	Specialist registrar, geriatrics

#### Ambiguity in stopping/de-escalating antibiotic therapy

Whilst junior physicians have a huge weight of expectation to start antibiotics as quickly as possible in patients suspected of having infection, the opposite appears true of them stopping or de-escalating therapy. A key factor throughout the interviews was that on-rotation doctors are not expected to stop or de-escalate therapy, with this responsibility seen as something only consultants and specialist registrar trainees undertake. Furthermore, it was widely reported by junior physicians that there is often variable feedback on the decisions that they have made following review and refinement by an external reviewer. This caused a great deal of frustration with junior prescribers, who often did not fully appreciate why their decisions had been over-ruled/changed and therefore feel that they do not develop a deep understanding of this skill. Another area of frustration reported by junior doctors was the heterogeneity between senior clinicians to how they approach stopping or de-escalating therapy, which when teamed with lack of feedback can often deter trainees from even attempting to make or suggest changes to therapy in this respect. This is something that was supported by senior participants, who reflected on the lack of an evidence base for lengths of treatment to support them acting as senior decision-makers (Table 4).

**Table 4:** Selected quotes surrounding participants experiences and expectations of de-escalating/stopping antibiotics

Expectations around stopping/de-escalating therapy		
1	“*We are responsible for everything on the ward as well as all the decisions and I think we’ve got these practices in place which make sure that the antibiotics are stopped at a particular time when they needed to be stopped*”	Consultant, haematology
2	“*I’m complete disempowered* [to stop antibiotics]*, completely because they’re so complicated and the consultants who know their patients have their own ways of prescribing. It’s very unusual that anyone would actually explain to you what they’re thinking. I think I’ve had one explanation which was like a ray of sunshine*”	On-rotation, renal
3	“*In terms of stopping antibiotics yeah, I think stopping antibiotics is a very nebulous thing in itself… it is pretty random and is not really a huge amount of evidence out there.... I feel very happy with making decisions as to whether to stop after three times, seven, ten days whatever. I don’t think that’s a big issue*”	Consultant, general medicine
4	“*So I feel quite, I wouldn’t say disempowered, but I feel like the seniors make most of the decisions. So I’m quite reluctant to make any decisions about* [de-escalating] *antibiotics*”	On-rotation, gastroenterology
5	“*Stopping them is generally, from my experience, has been a senior’s* [decision]”	On-rotation, acute medicine 1
6	*“De-escalating can be a little bit more tricky, it’s very much individually based.* [For] *some people it’s easier but if there’s no plan in place, if someone hasn’t said for five days, go for IVs and then deescalate to PO I would be hesitant. I would tend to want to get a little bit of reassurance”*	On-rotation, acute medicine 2

#### The role of guidelines and microbiology advice

Antimicrobial prescribing guidelines and clinical microbiology services play a large role in the decision-making process for infection management, despite senior physicians taking responsibility for the patients’ overall management and care. On-rotation and specialist trainee physicians report adherence to guidelines for prescribing as they realise that this is the expectation of their senior colleagues and the hospital. Consultants report that their job is to ensure that these guidelines are adhered to when this is appropriate, but also retain autonomy to be able to adapt guidelines based on their own experience and feel for the situation.

For on-rotation and specialist trainee physicians, microbiology services and advice is seen as a very valuable and convenient point of access, often referred to as a safety-net for challenging decisions, which are not necessarily outlined in the local antimicrobial guidelines or when a junior physician is not confident that they have selected the correct treatment. It is at this point that physicians tend to “*just call microbiology and ask….*”. However, several issues with the reliance on microbiology services for helping the decision-making process are also reported by senior physicians. These include poor communication pathways during microbiology discussions, the lack of microbiologist responsibility for outcomes of therapy recommended and a lack of continuity in the service provided due to rotation of trainee physicians. Furthermore, this perceived lack of responsibility reported means that consultants report that they are often reluctant to change decisions based on the advice of junior colleagues from other specialties, such as microbiology, especially when it is perceived that they are not fully aware of all the patient factors outlined in the decision process (Table 5).

**Table 5:** Selection of participant quotes surrounding their antimicrobial guidelines, clinical microbiology services and some problems associated with information provided by these sources

No.	Quote	
Reliance on guidelines		
1	“*Does that really change your management? With the majority of cases it hasn’t. So you strap them on the standard hospital protocol for CAP/infective exacerbation and you tend to just carry it on*”	On-rotation, acute medicine 1
2	“*Well because we’re almost held down now by* [antibiotic app guidelines] *or whatever your Trust uses, so you end up, if you haven’t done something by that choice you will go, or normally a pharmacist will go, why haven’t you done that?*”	On-rotation, acute medicine 2
3	“*I do find antibiotic guidelines very helpful, and actually in the last couple of trusts I’ve worked in, they’ve been so comprehensive that I’ve not really used any other sources at all*”	Specialist registrar, geriatrics
4	“*I think in terms of decision making I have to say I don’t keep up to date with the antibiotic formula because I look it up if I need it*”	Specialist registrar, cardiology
5	“*Quite often on a post-take ward round say, why are we giving this, has anyone checked the policy, is this in line with policy because I don’t think it is?*”	Consultant, respiratory
Reliance on microbiology		
1	“*If I think it clearly isn’t within guideline or I’m not sure, it doesn’t easily fit into the guideline I’m going to say, speak to micro*”	Consultant, respiratory
2	“*I think when you call the microbiologist the fact that you’ve made the call has already told them that you’re concerned so you’re almost saying, I want a change, give me further guidance*”	Consultant, geriatrics
3	“*If the patient has a lot of allergies for example, then that often makes it more difficult and I often end up speaking to micro if that’s the case*”	On-rotation, respiratory
Problems with guidelines and microbiology		
1	“*I mean I’m a complete pedant I hate this idea that microbiologists have just given antibiotics broad spectrum for sepsis of unknown origin because that’s not what I’m about as a physician”*	Consultant, gastroenterology
2	“*I think the difficult thing which sometimes arises that microbiology are often the more conservative end of the antibiotic spectrum and say, OK, you’ve had your course, stop and I may agree with that as a registrar. But the problem is that actually suggesting for me to do it is the wrong person because it’s my decision once I’ve seen the patient on the ward round, but once you’ve got a consultant* [microbilogist] *that’s come and ratified the decision then that becomes their decision*”	Specialist registrar, cardiology
3	“*They tend to give more of a patient specific approach but the difficulty in that is that they haven’t seen the patient. So they’re sort of just giving you advice over the telephone*”	On-rotation, gastroenterology
4	“*A lot of the time is I would maybe rather wait and speak to someone whose opinion and knowledge seems more valuable, where sometimes maybe the opinion that you get out of hours* [from junior microbiologists] *is someone who is just answering a question to get it dealt with, and so it’s too broad, it’s too much*”	Consultant, respiratory
5	“*Well it’s not patient specific* [local guidelines] *so it’s quite generalised and it won’t always have all the information about the patient*”	On-rotation, respiratory
6	“*I always think that people and especially microbiologists recommend changing antibiotics far too soon. You ring up a micro registrar who just says, oh immediately I want to change from Augmentin to Tazocin. Well, why?*”	Consultant, gastroenterology

## Discussion

Medical physicians report a common stepwise approach to the decision process surrounding acute infection management, where new information is constantly considered in the context of prior knowledge in a dynamic and often multi-level Bayesian-like process. Despite a common overall approach, a number of factors alter the weighting given to individual variables in this process, many of which focus on the later phases of the decision process (initiation of therapy and reviewing/refining those decisions). Factors that significantly influence this are expectations of providing optimal care for the patient, perceptions surrounding stopping and de-escalating therapy, and interactions with local guidelines and microbiology advice provided by specialists. Previously reported factors of team hierarchies and prescribing etiquette, also featured heavily [7, 29, 30]. Prescribing etiquette refers to the unwritten social code of practice around antibiotic prescribing which includes the desire for clinical autonomy and the reluctance to interfere with the antibiotic prescribing behaviours of peers [7].

### Windows of influence on decision making

There are four defined spheres of influence that affect physician-reported decision-making in the infection pathway. These could be thematically identified as either consciously or subconsciously influencing the non-infection specialist medical physician’s decision process for acute infection management. These are (1) implicit factors (such as the stages reported in Fig. 1), which are known to both the individual and the wider team and are what are commonly incorporated into guidelines and protocols for antimicrobial use such as the ‘start-smart-and-focus’ campaign within the UK [31]; (2) explicit factors, which are often blind spots that the individual often is not aware of but may be appreciated by others, such as observations of team hierarchies and prescribing etiquette; (3) internalised rationale (or hidden reasoning), that is known to the individual but often not externalised to others, such as is reported about senior decision-makers who do not feedback rationale for changing therapy to more junior colleagues (this can often cause confusion and frustration when reasons for decisions are not shared beyond the individual who has made them); and (4) subconscious influences that are neither identified by the individual or wider cohort, but are likely to play a significant role in the decision-making process. This could potentially include the role of other disciplines such as pharmacists and nursing staff who have been demonstrated to have a role in promoting optimal use of antimicrobials in several settings [32, 33], but were seldom reported and not identified by the participants in this study. This is especially grave, given that the role of the pharmacist, in the UK, is often described as the corner stone of antimicrobial stewardship (AMS) interventions and further highlights the need to challenge the current prescriber hierarchies that exist within the hospital setting [32, 34]. What could be observed from physician interviews was that a major factor for the engagement of pharmacists in the decision process appeared to be their level of seniority and presence and involvement in the core medical team caring for the patient.

“*And the pharmacists are often good, I think when we often have the pharmacists on the post-take ward round and it depends a bit on their seniority and confidence, so the ones who will speak up and challenge are excellent*” [Consultant, respiratory]

Therefore, interventions may also benefit from targeting the promotion of multi-professional integration to help normalise the role of the pharmacist and other healthcare professionals within the decision-making process surrounding infection management.

Despite there being several windows of influence that appear to contribute to the decision-making process, current interventions that include quality improvement and guidelines/policy appear to only focus on the initial implicit factors identified. Broader approaches to address the wider social and cultural knowns and unknowns must also be considered if we are to have a significant impact on the non-expert prescriber’s decision pathway. This is especially important as AMR is now a major driver of the patient safety and political agenda. With this, the role of behavioural sciences in promoting the appropriate and judicious use of antimicrobial agents has become a leading theme for AMS interventions [8, 34, 35]. The role of team dynamics and hierarchy has been explored in the intensive care unit and also considered for translation into clinical decision support software [30, 36–38]. However, our study has highlighted that simply understanding the decision process and incorporating it into AMS interventions is unlikely to be successful given the complex factors that influence decision making at all levels of the physician hierarchy.

### Distributing responsibility for decision making

A major theme emerging from this study was that of responsibility for the decisions that are made for the patient. This was highlighted when consultants considered clinical microbiology advice. Whilst the role of clinical microbiology was seen as a great help overall, senior clinicians often see the quality of the advisory services to be dependent on the information that is provided by junior colleagues, the lack of continuity in who they gain advice from, and the limited responsibility for the consequences of therapy that the microbiologist has when they provide advice. This links with the senior clinicians’ experience and autonomy in decision-making, which often leads to frustration and consideration of alternative treatments that may not be based on evidence. Therefore, it would seem that, to effectively address these perceived issues, some level of responsibility for the impact of prescribing decisions must be distributed beyond that of the senior consultant in charge of the patient.

### Addressing the role of antibiotics in providing optimal care

Physicians report reflective practices as they progress through their training. They report developing an understanding, that as junior trainees, they were scared of sepsis or under treating an individual and therefore causing harm. As a response to this concern, they focus solely on the short term, preferring to prescribe broad-spectrum agents and seek senior physician support to refine these decisions. This decision process is further supported by the provision of detailed local prescribing guidelines, which provide junior trainee’s with justification for making prescribing decisions and protecting them from judgement by their senior team members, even if those decisions are incorrect. This is further reinforced by the expectation placed upon them to be able to prescribe antibiotics for infections. The opposite is true of stopping or de-escalating antimicrobials, which is seen as a more serious decision that could affect the patient negatively and is therefore deferred to the senior decision-makers. To effectively promote improvements in antimicrobial use in secondary care these assumptions must be effectively challenged to address the negative aspects of antimicrobial therapy and empower individuals in revising the decisions that they have made.

### Limitations

This study had several limitations. Firstly, we only interviewed medical physicians working in a narrow number of specialties from one UK NHS hospital Trust, meaning that there may be variations in the team dynamics and workflows in different specialties (such as surgery) and regions of the world. Furthermore, the researcher, a junior medical doctor, performed all of the interviews (TMR), which was considered as a potential source of bias during the interview and analysis process. To address this during data analysis, a multi-professional group of researches involving doctors (LSPM, AH), a pharmacist (EC), a nurse (ECS), and lay researchers (BH, PH, PG) all reviewed the data and provided input on final thematic selection. Finally, although our theoretical sampling methodology followed validated guidelines and we purposefully sought out deviant statements to contradict emerging themes, the reliance on individual responses to invited emails may have introduced selection bias as individuals interested in antimicrobial prescribing and stewardship may have been more likely to respond to invitations [25, 28].

## Conclusion

In conclusion, we have identified that physicians in secondary care adopt a Bayesian approach to the decision process for infection management. Whilst a large number of factors influence how physicians weight individual variables, there is also a common theme, which must be addressed if behaviour change interventions promoting optimised antimicrobial prescribing are to be successful. These include distribution of the responsibility of prescribing decision, fostering an earlier understanding of the risks of antimicrobial therapy and expectations about de-escalation, and promotion of true multi-professional involvement in decision-making for infection management. Future studies must look to quantify the influence of identified variables on the decision-making pathway. Furthermore, reported decision-making pathways must be linked with observations from clinical practice to allow triangulation of reported findings and identification of further areas for targeted interventions to promote the optimal management of acute infections within secondary care.

## Appendix

Baseline questions used for decision mapping interviews

1. For a patient with an infection, describe how investigation and management decisions (including prescribing antimicrobials) are made.
2. If you had to rank (i) patient physiology, (ii) biomarker changes and (iii) microbiology results in their importance in relation to antimicrobial prescribing, how would you do so?
  *Prompts: how do these three factors relate to: sending further tests? If so which? Starting antimicrobials/narrowing spectrum of antimicrobials/stopping antimicrobials?*
3. How is the final decision on infection management made?
  *How are these decisions made out of hours at night? And at the weekend?*
4. Do you have a vision of an ideal way for infection management decisions to be made?
5. Are there any barriers to you making what you think are the optimal antimicrobial decisions?
6. Are there any aids that help with making the optimal antimicrobial decisions?
7. How do you access patient data when you are making infection management decisions?
8. How do you access published medical literature to help make decisions?
9. When do you access published medical information to help make decisions?
10. Do you feel empowered or dis-empowered to make decisions to start, stop or change antimicrobial prescriptions?

## Abbreviations

AMR: Antimicrobial Resistance
AMS: Antimicrobial Stewardship
